# Evaluating the prevalence and opportunity for technology use in chronic kidney disease patients: a cross-sectional study

**DOI:** 10.1186/s12882-018-0830-8

**Published:** 2018-02-02

**Authors:** Ann Bonner, Kerri Gillespie, Katrina L. Campbell, Katina Corones-Watkins, Bronwyn Hayes, Barbara Harvie, Jaimon T. Kelly, Kathryn Havas

**Affiliations:** 1 School of Nursing, Queensland University of Technology, Brisbane, Australia; 2 Kidney Health Service, Royal Brisbane and Women’s Hospital, Herston, Australia; 3 NHMRC Chronic Kidney Disease Centre of Research Excellence, University of Queensland, Herston, Australia; 4 Renal Service, Princess Alexandra Hospital, Woolloongabba, Australia; 5 Faculty of Health Sciences and Medicine, Bond University, Gold Coast, Australia; 6 School of Nursing and Midwifery, Griffith University, Gold Coast, Australia; 7 Renal Unit, Cairns Hospital, Cairns, Australia; 8 Renal Unit, Queanbeyan Hospital, Queanbeyan, Australia

**Keywords:** Mobile phone, Internet, Smart phone, Telehealth, E-health, mHealth, Chronic kidney disease, End stage kidney disease

## Abstract

**Background:**

Chronic kidney disease (CKD) is increasing worldwide and early education to improve adherence to self-management is a key strategy to slow CKD progression. The use of the internet and mobile phone technologies (mHealth) to support patients is considered an effective tool in many other chronic disease populations. While a number of mHealth platforms for CKD exist, few studies have investigated if and how this population use technology to engage in self-management.

**Methods:**

Using a cross-sectional design across five health districts in Queensland (Australia), a 38-item self-report survey was distributed to adults with CKD attending outpatient clinics or dialysis units to measure current use and type of engagement with mHealth, perceived barriers to use, and opportunities to support CKD self-management. Odds ratio (OR) were calculated to identify associations between demographic characteristic and mHealth use.

**Results:**

Of the 708 participants surveyed, the majority had computer access (89.2%) and owned a mobile phone (83.5%). The most likely users of the internet were those aged ≤ 60 years (OR: 7.35, 95% confidence interval [CI]: 4.25–12.75, *p* < 0.001), employed (OR: 7.67, 95% CI: 2.58–22.78, *p* < 0.001), from non-indigenous background (OR: 6.98, 95% CI: 3.50–13.93, *p* < 0.001), or having completed higher levels of education (OR: 3.69, CI: 2.38–5.73, *p* < 0.001). Those using a mobile phone for complex communication were also younger (OR: 6.01, 95% CI: 3.55–10.19, *p* < 0.001), more educated (OR: 1.99, 95% CI: 1.29–3.18, *p* < 0.01), or from non-indigenous background (OR: 3.22, 95% CI: 1.58–6.55, *p* < 0.001). Overall, less than 25% were aware of websites to obtain information about renal healthcare. The mHealth technologies most preferred for communication with their renal healthcare teams were by telephone (56.5%), internet (50%), email (48.3%) and text messages (46%).

**Conclusion:**

In the CKD cohort, younger patients are more likely than older patients to use mHealth intensively and interactively although all patients’ technology literacy ought to be thoroughly assessed by renal teams before implementing in practice. Further research testing mHealth interventions to improve self-management in a range of patient cohorts is warranted.

**Electronic supplementary material:**

The online version of this article (10.1186/s12882-018-0830-8) contains supplementary material, which is available to authorized users.

## Background

Self-management requires individuals to know how to monitor their disease, manage symptoms, interpret results of home-monitoring therapies, and to carry out daily treatment plans including adhering to complex medication regimens, dietary and fluid restrictions, and for some, perform dialysis at home. To enable people with CKD do this, renal clinicians (and others) use a range of educational and behavioural strategies to support self-management behaviours. However, clinicians largely believe that if people have adequate knowledge about what to do, they will engage in self-management behaviours [1]. This is problematic because adequate understanding of CKD and its management remains bewildering for many who are attending specialist renal services [2–5].

Technologies such as smart phones, tablets, laptops, and other transportable devices are being increasingly used to deliver information and educational support programs to patients. eHealth includes a wide range of devices and technologies (including websites, robotics, alarms, and communication platforms) for the monitoring, detection, management and treatment of individuals [6]. A component of eHealth, ‘mHealth’ has more recently been coined as a term describing the use of mobile and wireless devices, exploiting the ability to speak and message in real time [7]. Phone ‘Apps’ as a type of mHealth have also become valuable tools as medication alarms and reminders, monitoring devices, and scheduling and education delivery systems [8]. The millennial term, ‘digital divide’, refers to the socioeconomic or age-related inequality of internet access and technical literacy [9]. This is a serious issue impinging on the use of mHealth, as many technologies assume levels of computer knowledge and unrestricted internet access that may not exist.

Internationally there are a number of educational websites to inform people about CKD and its risk factors, treatment options along the course of the disease trajectory, as well as related diseases [6, 10]. However, those with CKD are less likely than healthy adults to access the internet [11, 12] or even know that information is available [2, 6]. Determinants to internet access in the CKD population include: access to a computer, technological literacy, and income and education levels [10, 11]. In those receiving haemodialysis (HD), previous studies show that access the internet is 35% and 58% in the United States and Canada respectively [13, 14]. Moreover, clinicians tend to over-estimate patient access to and use of these technologies to manage their health [15, 16].

The use of and potential for mHealth has not been established in the Australian CKD population. Prior to developing mHealth applications for this group of patients, this study sought to understand whether and how individuals were accessing and using mobile phones and the internet; and whether they used the internet to gather any renal information. Knowledge of these determinants will help identify the most effective education platforms for this patient population.

## Methods

Using a cross-sectional design, this study was conducted at five renal services (2 regional, 3 metropolitan) in Queensland, Australia. Inclusion criteria were: > 18 years; attending a CKD outpatient clinic (regardless of CKD stage) or dialysis unit with end-stage kidney disease (ESKD); and able to read and write English, or have a family member who could assist with the completion of the survey. People with a cognitive impairment, limited English language ability or who had a functioning kidney transplant were excluded from the study. Data was collected over a nine-month period between June, 2015 and March, 2016.

### Sample size

Utilising the most conservative ‘rule of thumb’, 20 participants per item determined the study sample size [17, 18]. This study therefore required a minimum of 380 participants.

### Instrument

Data was collected using a 38-item survey which assessed factors associated with internet and mobile phone use; barriers to access; types of information accessed; and why and how information is accessed (see Additional file 1). It was developed by the researchers after a thorough investigation of the literature revealed important topics and issues relating to patients’ information needs and technology use. Items were a mixture of multiple choice and short answer; such as “How often do you access the internet?”; “Have you ever used the internet to find information about your kidney health condition?”; “Do you use your mobile phone for any of the following activities?” The survey was designed to be easily understood and completed by those with low literacy levels (accessible to those at a primary school reading level ability or above). It was first tested on a sample of ten patients (who were not part of the main study) who reported no difficulty in responding to items; it was then implemented without alteration. Self-reported demographic characteristics (age, gender, level of education, employment status, and postcode) were also collected. Due to the high prevalence of CKD in the Australian indigenous population [19], participants were also invited to indicate whether they identify themselves as ‘Aboriginal’, ‘Torres Strait Islander’, or both, and have therefore been grouped under the classification ATSI (Aboriginal and/or Torres Strait Islander). Surveys were completed in the waiting room of the renal outpatient clinic or dialysis unit and returned by placing in a secure box or completed at home and returned in reply-paid envelopes.

### Data analysis

Statistical analysis was performed using IBM SPSS Version 23.0 [20]. For analysis we grouped age into 18–40, 41–50, 51–60, 61–70 and > 71 years. Education level was divided at those who did and did not complete high school, as year 12 completion is an indicator of continuing further education [21]. Categorical variables were described using frequencies and percentages, and bivariate relationships were explored using chi-square analysis. We defined mobile phone use as: i) simple (only making voice calls and short message service [SMS] sending and retrieval); ii) complex (simple use plus sending photos/videos, playing music/games, using phone calendar); and iii) complex apps (use of phone applications such as banking, social media, email, skype, online bookings, shopping).

Binary logistic regression was used to determine strength and direction of relationships and to allow for adjustments of demographic variables (age; gender; education; ethnicity; employment; and remoteness [determined by postcode]). Odds ratios (OR) and 95% confidence intervals (CI) were reported. Multivariable models, to identify factors influencing the use of mobile technologies and health information access, were created using forward stepwise modelling using demographic variables. This modelling technique was chosen based on the number of variables, and the high likelihood of multicollinearity in this subset of variables. A significance level of *p* < 0.05 was used.

## Results

Initially 720 surveys were collected across all sites although 12 (1.7%) were excluded due to incompleteness (< 80% of questions were answered, and/or crucial questions relating to internet and mobile phone use, fundamental to the research question, remained unanswered). In total, 708 completed surveys were used in the final analysis. Just over half of the sample was male (55.2%) and about half were aged > 61 years (51.6%). The largest portion was 71 years of age and over (29.3%). The majority of participants (83.1%) were not Aboriginal or Torres Strait Islander (non-ATSI), while 11.7% self-identified as ATSI. See Table 1 for demographic characteristics.

**Table 1:** Participant demographics

		Number	Percent
Site			
	Site 1 (Metropolitan)	243	34.3
	Site 2 (Metropolitan)	137	19.4
	Site 3 (Metropolitan)	143	20.2
	Site 4 (Regional)	104	14.7
	Site 5 (Regional)	81	11.4
Gender			
	Male	389	54.9
	Female	316	44.6
	Missing	3	0.4
Age			
	18–40	119	16.8
	41–50	108	15.3
	51–60	115	16.2
	61–70	158	22.3
	71+	207	29.2
	Missing	1	0.1
Ethnicity			
	ATSI	78	11.0
	Non-ATSI	588	83.1
	Prefer not to indicate or missing	42	5.9
Employment			
	Employed (F/T and P/T)	161	22.8
	Unemployed	36	5.1
	Pensioner / Retired	439	62.2
	Other (Student/home duties)	70	9.9
	Prefer not to indicate or missing	2	0.3
Education level			
	Did not complete high school	346	48.9
	Year 12 or equivalent	345	48.7
	Prefer not to indicate or missing	17	2.4
Region / remoteness			
	Major City	451	65.3
	Inner Regional	129	18.7
	Outer Regional and remote	111	16.1
	Prefer not to indicate or missing	17	2.4
Currently receiving dialysis			
	Yes	269	38.5
	No	429	61.5
Type of dialysis			
	Haemodialysis in hospital or satellite unit	219	81.4
	Haemodialysis in the home	26	9.7
	Peritoneal dialysis in the home	18	6.7
	Missing	6	2.2

*ATSI* aboriginal and Torres Strait Islander, *F/T* full-time, *P/T* part-time

### Mobile phone use

Most of the sample owned a mobile phone (*n* = 588, 83.5%); with most reporting that it was a smart phone (*n* = 378, 64.3%) although 4.9% (*n* = 29) were unsure. More than half (*n* = 456, 77.6%) could use the phone for complex activities although this was mostly due to taking photos. Fewer indicated that they could use mobile phone apps (*n* = 215, 36.6%). Mobile phone ownership was significantly more common in those ≤60 years of age (*p* < 0.01), employed (*p* < 0.01), and those with higher education levels (*p* < 0.01). There were no differences for either ethnicity or whether or not on dialysis (see Table 2).

**Table 2:** Mobile phone ownership within demographic groups; chi-square analyses

Demographic Characteristics		Own A Mobile Phone Frequency (%)	Do Not Own Mobile Phone Frequency (%)	*p*
Age^a^ (*n* = 703)				
	60 and under	320 (94.1)	20 (5.9)	< 0.01
	61 and over	267 (73.6)	96 (26.4)	
Ethnicity^a^ (*n* = 664)				
	ATSI	60 (76.9)	18 (23.1)	0.11
	Non-ATSI	493 (84.1)	93 (15.9)	
Employment^a^ (*n* = 702)				
	Employed	156 (97.5)	4 (2.5)	< 0.01
	Retired/unemployed	430 (79.3)	112 (20.7)	
Education^a^ (*n* = 687)				
	Did not complete high school	271 (78.6)	74 (21.4)	< 0.01
	Grade 12 and over	305 (89.2)	37 (10.8)	
Dialysis^a^ (*n* = 694)				
	On dialysis	226 (84.3)	42 (15.7)	0.67
	Non-dialysis	354 (83.1)	72 (16.9)	

*ATSI* Aboriginal and Torres Strait Islander ^a^ analysis conducted on available data

**Fig. 1:**
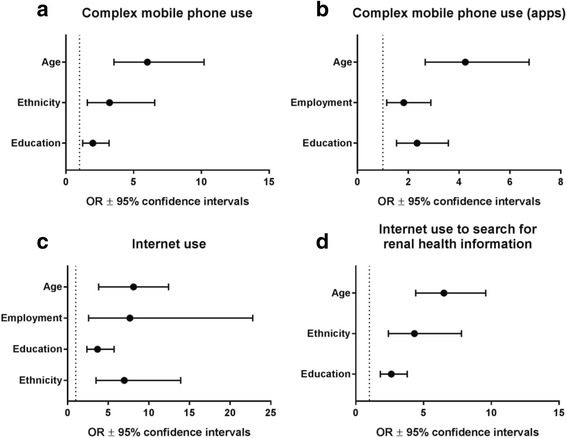
Logistic regression model for odds of mobile phone and internet use. Odds ratios ±95% confidence intervals derived from logistic regression for **a**) complex mobile phone use; **b**) complex mobile phone use (apps); **c**) internet use; and **d**) internet use to search for renal health information. Dashed line at x = 1 indicates referent group (age > 61 years; ethnicity = ATSI; education = less than 12 years of schooling; employment = retired/unemployed)

Multivariate binary logistic regression showed significant relationships between complex use and age, ethnicity, and education levels (see Fig. 1a). Participants aged ≤60 had over six times the odds of using their mobile phone for more complex tasks than did those aged 61 and over (OR: 6.01, CI: 3.55, 10.19, *p* < 0.001). Non-ATSI participants had over three times the odds of complex phone use than did those who identified as ATSI (OR: 3.22, CI: 1.58, 6.55, *p* = 0.001). Those who had obtained higher levels of education had almost double the odds of complex use (OR: 1.99, CI: 1.24, 3.19, *p* = 0.004).

Approximately one third (29.5%) of participants reported that they had downloaded at least one mobile phone application (e.g. Twitter, banking) in the past month. When examining the complex apps use as a demonstration of greater mHealth literacy, a similar relationship was demonstrated between app use, age, education, and employment; though employment was seen to be more of a predictor than ethnicity (see Fig. 1b). Younger participants (≤ 60 years) had over four times the odds of complex app use (OR: 4.25, CI: 2.67, 6.76, *p* < 0.001), while those who were employed (OR: 1.83, CI: 1.15, 2.89, *p* = 0.01) and those who had obtained higher levels of education (OR: 2.35, CI: 1.54, 3.58, *p* < 0.001) had approximately twice the odds of complex app use.

#### Internet use

Of the total cohort who reported using the internet (*n* = 491, 69.4%), most used it at home (*n* = 379, 89.4%) for more than 60 min per day (*n* = 252, 51.9%) through a laptop (*n* = 113, 32.3%) or desktop computer (*n* = 107, 30.6%). The most frequently reported internet activities were checking emails (*n* = 395, 80.9%), searching/browsing the internet (*n* = 366, 74.5%), accessing social networks (*n* = 245, 50.2%), accessing health information (*n* = 221, 45.3%), and reading/watching the news (*n* = 217, 44.5%). A total of 217 participants (30.6%) reported that they did not use the internet, of those more than half (*n* = 118, 54.4%) reported that they did not know how to use the internet. A large number of participants also indicated that hospitals should provide free access to WiFi (*n* = 464, 70.3%).

Binary logistic regression modelling (see Fig. 1c) indicated that four factors impacted on people’s odds of having used the internet: age (participants ≤ age 60 had over seven times the odds of having used the internet, OR: 7.36, CI: 4.25, 12.75, *p* < 0.001); employment (OR: 7.67, CI: 2.58, 22.78, *p* < 0.001); education (OR: 3.69, CI: 2.38, 5.73, *p* < 0.001); and ethnicity (OR: 6.98, CI: 3.50, 13.93, *p* < 0.001).

#### Searching the internet for information related to CKD

Just under half of the sample (*n* = 332, 49%) reported that they had used the internet to seek specific information regarding their CKD. Only 189 participants (27.4%) reported that their family had sought information about CKD on the internet on request. Those seeking health information online were younger (*p* < 0.01), less likely to be indigenous (*p* = 0.03), and more likely to be employed (*p* < 0.01) and have obtained higher educational qualifications (*p* < 0.01). There was no difference between those who were or were not receiving dialysis (see Table 3).

**Table 3:** Participants who use the internet to search for renal health information; chi-square analyses

Demographic characteristics		Used the internet to seek renal health information Frequency (%)	Did not use the internet to seek renal health information Frequency (%)	*p*
Age^a^ (*n* = 675)				
	60 and under	235 (70.1)	100 (29.9)	< 0.01
	61 and over	97 (28.5)	243 (71.5)	
Ethnicity^a^ (*n* = 640)				
	ATSI	25 (33.8)	49 (66.2)	0.03
	Non-ATSI	296 (52.3)	270 (47.7)	
Employment^a^ (*n* = 674)				
	Employed	114 (72.2)	44 (27.8)	< 0.01
	Retired/unemployed	218 (42.2)	298 (57.8)	
Education^a^ (*n* = 662)				
	Did not complete high school	110 (33.6)	217 (66.4)	< 0.01
	Grade 12 and over	219 (65.4)	116 (34.6)	
Dialysis^a^ (*n* = 667)				
	On dialysis	127 (50.4)	125 (49.6)	0.71
	Not on dialysis	203 (48.9)	212 (51.1)	

*ATSI* Aboriginal and Torres Strait Islander ^a^ analysis conducted on available data

As age increased, there was a significant reduction in the use of the internet to seek health information regarding kidney ‘problems’ (renal impairment); from 78.8% ≤ age 40), decreasing steadily to 19.1% (≤ age 70; χ2(4) = 137.389, *p* < 0.01). Older participants were less likely to have known of any websites for kidney patients, though no age group had an explicit knowledge of these sites (37.9% of those ≤40, decreasing to 10.8% of those > 71 (χ2(4) = 44.723, *p* < 0.01). Participants were also more likely to have heard of kidney websites if they were on dialysis (31.9% versus 19.7%; χ2(1) = 12.867, *p* < 0.01).

Figure 1d illustrates the impact of age, ethnicity and education on searching the internet for renal health information. Younger participants had 6.5 times the odds of having used the internet to seek for renal health information (OR: 6.51, CI: 4.42, 9.59, *p* < 0.001). Non-ATSI and more educated participants had over 4 times the odds (OR: 4.33, CI: 2.41, 7.79, *p* < 0.001) and 2.6 times the odds (OR: 2.62, CI: 1.81, 3.78, *p* < 0.001), respectively.

#### Facilitators and barriers to mHealth

When participants were asked what mHealth technologies they would be willing to use to engage with their healthcare team, the most common modalities indicated were telephone calls (*n* = 400, 56.5%), followed by the internet (*n* = 354, 50%), email (*n* = 342, 48.3%) and SMS messages (*n* = 326, 46%; note: participants could indicate more than 1 modality). Overall the perceived barriers to using technologies were low although the barrier ‘do not know how to use’ was indicated more frequently for SMS messages (*n* = 94, 13.3%), emails (*n* = 112, 15.8%) and visiting a website (*n* = 108, 15.2%).

## Discussion

To our knowledge, this is the first study to identify the use of mHealth in an Australian population of CKD patients. We found that patients older than 60 years, those from an ATSI background, and those with lower levels of education (did not complete secondary school) were engaging in only simple communications functions with their mobile phones. Whereas younger, more educated and employed patients could undertake more sophisticated activities with their mobile phones and were also more likely to use the internet. Our findings also indicated that, regardless of age, the level of access to technology (including mobile phone ownership) was high. While new technologies for mHealth are being developed rapidly to improve education, treatment, and service delivery for patients with a range of chronic diseases [6], our findings indicate that a large number of patients with CKD will be alienated from and excluded if there is a uniform move to adopting these technologies.

In other chronic diseases such as heart failure, diabetes and chronic obstructive pulmonary disease (COPD), mobile phones are frequently used to deliver patient education and self-management support [22–24]. When utilised, mHealth (reminder services, systems, or booking systems) can have beneficial impacts on health care costs, self-efficacy, and clinical outcomes such as blood pressure and glycated haemoglobin A1c [24–26]. Although a number of studies have described the benefits of these interventions, several systematic reviews have found that the results of these studies were small or inconclusive [22, 24, 27–29]. High attrition rate is the major issue facing many studies investigating technologies, with up to 78% of participants failing to use, or rarely using the technologies being investigated [27, 30]. The common pattern of attrition in these studies has often been explained by technological difficulties faced by participants, the increased time burden of technology interventions, and costs involved in maintaining these services [31, 32]. Nevertheless patient-reported barriers to mHealth tend to be due to technical problems or having an aversion to using technology, and that is still a preference for face-to-face interaction with health care providers [27]. Simple, less time-consuming technology which is user-friendly is more likely to be acceptable in those with chronic disease [33].

Previous studies in kidney transplant recipients [9], and other chronic diseases [15, 34] have also found that sociodemographic characteristics (age, education level, ethnicity) influences internet use. Our study found a low prevalence of barriers related to a lack of knowledge on computer use, disinclination or unwillingness to use, and no access to a computer, reflecting the potential for a digital divide in this population. These reasons are comparable to the 2014–15 Australian Bureau of Statistic (ABS) figures; reporting the main reasons for no computer access as no need (63%), lack of confidence or knowledge (22%), and cost (16%) [35]. Access to technology is associated with higher socioeconomic levels (e.g. level of education and income. Park [36] argues that there is a ‘double jeopardy’ of social exclusion and geographical remoteness contributing to the digital divide in Australia. Regardless of location, the digital divide is further exacerbated because the acquisition of digital literacy skills are either not acquired or developed alongside the growth of technology in everyday life. For those not using these technologies, traditional methods of education and service delivery ought to be retained.

Another possibility for low adoption of online services and sites is that they are not tailored to suit the general public’s digital and health literacies. Being able to locate and navigate online sites can be challenging, and then layering on the need to comprehend CKD-specific information adds further complexity. CKD websites and YouTube vary greatly in the amount and type of information provided, and these are rarely written at a literacy level easily understood by the average CKD patient [37–39]. In a review of eHealth studies, Irizarry [40] found that higher health literacy capability contributed to the greater use of technology, indicating that those not participating in online services or programs would benefit the most from additional face-to-face information and support. Our results contribute to the importance of including CKD patients when developing mHealth strategies. This approach is likely to improve useability and uptake by a wider group of patients because the language, navigation, interface, and content, reflect a broader range of digital and health literacy capabilities [40].

There are currently a wide variety of online and mHealth interventions, ‘apps’, monitoring and communication services, educational websites, forums, patient portals and many more. There is no one superior model for mHealth delivery and clinicians ought to select the one most appropriate to their patient population. Ease of use for the patient (and clinician) is likely the most critical feature that dictates uptake and use of these technologies [32]. The findings for our study indicate that while many participants would be willing to use online sources and services for their care, capability must be thoroughly assessed prior to implementing as a strategy for supporting self-management.

### Limitations

The strength of this study was that the large sample included both non-dialysis and receiving dialysis, those attending renal services in both metropolitan and regional areas, and ATSI people with CKD. The non-dialysis group in this study were similar in age, gender and ethnicity to those in the CKD Queensland registry [41]. For those who were receiving dialysis age, ethnicity, and mode of haemodialysis were consistent with the Australian ESKD population in the Australian and New Zealand Dialysis and Transplant registry although this study had fewer peritoneal dialysis patients [42]. However, there are limitations of this study. First, kidney transplant recipients were excluded from this study warranting further research for this group of patients. Second, the instrument could have been designed with either additional age groups (e.g. 81+, etc) or capture a date of birth. Lastly, data was all self-reported and subjected to recall bias. As CKD is more prevalent in older adults and also the greatest increase in dialysis treatment is in the groups aged 65 and over in Australia [42], further research focussing on the barriers and facilitators of using mHealth in both the dialysis and non-dialysis groups are needed.

## Conclusion

Mobile phone ownership is high across age, education and socioeconomic status in Australia. Simple one-way SMS messages are likely to reach and be read by those with CKD and therefore would be useful for short, simple reminders to support self-management. Younger people, particularly with earlier stages of CKD, would benefit from more complex mHealth strategies that focus of primary prevention or to improve adherence with treatment. This study also offers a cautionary note as this patient population is not homogenous with respect to mHealth literacy and a one size fits all (or most) approach is unlikely to work. Thorough assessment of technology literacy by renal teams before deciding on education formats is advisable. There is still a vital role for face-to-face education and support for CKD patients. Further research of interactive mHealth support strategies, developed with both technology and health literacies in mind could increase the adoption by a wider group of patients.

## Additional file

Additional file 1: Telehealth survey instrument. (PDF 162 kb)

## Abbreviation

ATSI: Aboriginal or Torres Strait Islander
CI: Confidence interval
CKD: Chronic kidney disease
ESKD: End-stage kidney disease
mHealth: Internet and mobile phone technologies
non-ATSI: Not aboriginal or Torres Strait Islander
OR: Odds ratio
SMS: Short message service
