# Supplementary material for: Evaluating the prevalence and opportunity for technology use in chronic kidney disease patients: a cross-sectional study
Source: BMC Nephrol. 2018 Feb 2;19:28. doi: 10.1186/s12882-018-0830-8 (PMC5797344; doi:10.1186/s12882-018-0830-8)
Supplement: Additional file 1: — Telehealth survey instrument. (PDF 162 kb) [file 12882_2018_830_MOESM1_ESM.pdf]

Queensland University of Technology
Brisbane Australia

Queensland Government

Instructions: Please tick whichever box applies to you.

Part One: Demographic Information

1. Gender

☐ Male
☐ Female

2. Age Group

☐ 18-25 years	☐ 26-40 years	☐ 41-50 years
☐ 51-60 years	☐ 61-70 years	☐ 71-80 years
☐ 81-90 years	☐ Over 90 years	

3. Please state your ethnicity:

☐ Aboriginal
☐ Torres Strait Islander
☐ Aboriginal & Torres Strait Islander origin
☐ Neither Aboriginal/Torres Strait Islander origin

4. What is your postcode: ___________________

5. What is your employment status?

☐ Employed full-time
☐ Employed part-time
☐ Unemployed
☐ Pensioner/retired
☐ Student
☐ Home duties
☐ Other

6. What is the highest level of education you have achieved?

- ☐ Did not complete primary school
- ☐ Primary school only
- ☐ High School less than Grade 10
- ☐ High School Grade 10
- ☐ High School Grade 12
- ☐ TAFE
- ☐ College
- ☐ University

7. How long have you known that you have kidney problems?

- ☐ Less than 3 months
- ☐ 3 to 6 months
- ☐ 6 - 12 months
- ☐ Greater than 12 months

8. Are you currently receiving dialysis treatment?

- ☐ Yes → go to Q9
- ☐ No → go to Q10

9. If yes, please choose the type of your dialysis treatment

- ☐ Haemodialysis treatment in hospital or satellite dialysis unit
- ☐ Haemodialysis treatment at home
- ☐ Peritoneal dialysis treatment at home
- ☐ Other

Part Two: Internet Use

10. Have you ever used the internet?

- ☐ Yes → go to Q11
- ☐ No → go to Q18

11. How often do you access the Internet?

- ☐ Weekly or more
- ☐ Approximately twice a month
- ☐ Only once a month
- ☐ Once every 6 months
- ☐ Almost never

12. For how long have you been using the Internet?

- ☐ For less than 6 months
- ☐ Between 6 months and a year
- ☐ For more than a year

13. On average, how much time do you spend using the internet in a single day?

- ☐ Less than 30 minutes
- ☐ Between 30 minutes and 1 hour
- ☐ More than 1 hour

14. Where do you use the internet most?

- ☐ Home
- ☐ School, TAFE, or university
- ☐ At work
- ☐ At a friend's home
- ☐ At a relative's home
- ☐ Other public places (library, shopping centre, café etc)
- ☐ Other, please specify ______________________________

15. Which of these devices do you MAINLY use to access the internet?

- ☐ A smart phone (e.g. iPhone, blackberry)
- ☐ Laptop
- ☐ Tablet (e.g. iPad)
- ☐ Desktop computer
- ☐ A Television set (TV)
- ☐ A games console (e.g. PlayStation, Xbox)
- ☐ Other handheld personal devices (e.g. MP3 player, Ipod touch)
- ☐ Other, please specify_____________________________

16. Which of the following things have you done in the PAST MONTH on the internet? (Tick all that apply)

- ☐ Accessed health information
- ☐ Shopped for something
- ☐ Searched or browsed the internet
- ☐ Accessed chat rooms
- ☐ Checked email
- ☐ Gambled
- ☐ Listened to or downloaded music (e.g. itunes)
- ☐ Used online or email counselling
- ☐ Played games
- ☐ Social network (Twitter, Facebook etc)
- ☐ Read or watched the news
- ☐ Watched or downloaded movies, TV programs, Youtube)

17. Do you currently have easy access to a computer with an internet connection?

- ☐ Yes
- ☐ No

18. If you do **NOT** use the internet, are there some factors that stop you from using it?

- ☐ Don't know how to use the internet
- ☐ Don't want to
- ☐ No computer available
- ☐ Other reasons, please specify ______________________

19. Have you ever used the internet to find information about your kidney health condition?

- ☐ Yes
- ☐ No

20. Have you ever asked others (a family member or friend) to look up information about kidney health for you on the internet?

- ☐ Yes
- ☐ No

21. Do you think hospitals/dialysis units should provide wifi/internet access?

- ☐ Yes
- ☐ No

22. Do you think hospitals/dialysis units should provide computers, laptops or iPads for patients to use?

- ☐ Yes
- ☐ No

23. If hospitals/dialysis units provide computers in the waiting room, would you like to use it?

- ☐ Yes
- ☐ No

24. Have you heard of any internet websites for kidney patients?

- ☐ No
- ☐ Yes, please list any internet websites you know of to help manage your kidney condition

____

____

____

Part Three: Mobile Phone Use

25. Do you currently own and use a mobile phone?

- ☐ No → go to Q32
- ☐ Yes, what type of phone do you use (brand/model name)?

____

26. Is your mobile phone a 'smart phone' (i.e. can it be used to access the internet?)

- ☐ Yes
- ☐ No
- ☐ Don't know

27. What type of mobile plan do you have?

- ☐ Pre-paid (calls, text messages ONLY)
- ☐ Pre-paid (calls, text messages AND data package)
- ☐ Post-paid or contract (calls, text messages ONLY)
- ☐ Post-paid or contract (calls, text messages AND data package)
- ☐ Not sure

28. Do you use your mobile phone for any of the following activities? (Tick all that apply):

- ☐ Sending SMS (text messages)
- ☐ Receiving SMS (text messages)
- ☐ Make voice calls (to local, interstate, international and/or other mobile phone numbers)
- ☐ Make video calls
- ☐ Take photos
- ☐ Send and/or upload photos
- ☐ Take videos
- ☐ Send and/or upload videos
- ☐ Search or browse the internet
- ☐ Directions, maps and/or GPS functions
- ☐ Take notes
- ☐ Play games
- ☐ Calendar or diary function
- ☐ Use Apps
- ☐ Play music
- ☐ Make voice recordings (sound only)
- ☐ Set alarms and/or reminders
- ☐ Other, please specify:______________________

29. How many times in the LAST MONTH have you downloaded a mobile application?

- ☐ 0
- ☐ 1
- ☐ 2
- ☐ 3
- ☐ 4
- ☐ 5
- ☐ 6+

30. What were the last 3 mobile apps that you have downloaded?

1 ____

2 ____

3 ____

31. Please list which 3 mobile apps you use the most

1 ____

2 ____

3 ____

Part Four: Education about Kidney Health

32. What do you most want to learn about to help manage your kidney disease

____

____

33. Have you made a lifestyle change in the last 5 years?

- ☐ No
- ☐ Yes
- ☐ Don't know

If yes, what helped in making a lifestyle change?

____

34. Are you willing to use any of the following technologies to help you to learn more, develop skills, engage with your health care providers for kidney disease management? (Tick all that apply):

- ☐ Phone (calls)
- ☐ Phone (SMS)
- ☐ Email
- ☐ Website (to access information)
- ☐ Video (DVD or CD)
- ☐ Videoconference (e.g. WebEx, Skype)

35. If not, what do you believe are barriers to using these technologies (place a 'X' in the box where it applies):

	**Phone (calls)**	**Phone (text /SMS)**	**Email**	**Visiting a website**	**Video (DVD or CD)**	**Video-conference**
Do not know how to use						
Do not like to use						
Not private						
Do not think it's useful						
Not secure						
Prefer to talk face-to-face						
No time						
Other						

Part Five: Photographic Record of Intake

36. Have you ever had to measure or keep a record of your diet or been asked to recall the foods you eat?

- ☐ Yes
- ☐ No

37. Would you be willing to take photographs of the food you eat using your mobile phone, or other device if provided

- ☐ Yes
- ☐ No → What would be some of the reasons that you would not use your phone (or other device) in this way:

____

____

38. What is the maximum period of time that you think you would be able to photograph your diet using your mobile phone?

- ☐ 1 day
- ☐ 3 days
- ☐ 5 days
- ☐ 7 days
- ☐ 14 days
- ☐ 30 days
- ☐ As long as required

Thank you! Your participation is greatly appreciated ☺
